# Analysis of a Routinely Used Commercial Anti-Chikungunya IgM ELISA Reveals Cross-Reactivities with Dengue in Brazil: A New Challenge for Differential Diagnosis?

**DOI:** 10.3390/diagnostics11050819

**Published:** 2021-04-30

**Authors:** Monique da Rocha Queiroz Lima, Raquel Curtinhas de Lima, Elzinandes Leal de Azeredo, Flavia Barreto dos Santos

**Affiliations:** Viral Immunology Laboratory, Oswaldo Cruz Institute, Rio de Janeiro 21040-900, Brazil; raquel.lima@ioc.fiocruz.br (R.C.d.L.); elzinandes@ioc.fiocruz.br (E.L.d.A.)

**Keywords:** chikungunya diagnosis, anti-chikungunya IgM ELISA, dengue cross-reactivity, Brazil

## Abstract

In Brazil, chikungunya emerged in 2014, and by 2016, co-circulated with other arbovirosis, such as dengue and zika. ELISAs (Enzyme-Linked Immunosorbent Assays) are the most widely used approach for arboviruses diagnosis. However, some limitations include antibody cross reactivities when viruses belong to the same genus, and sensitivity variations in distinct epidemiological scenarios. As chikungunya virus (CHIKV) is an alphavirus, no serological cross reactivity with dengue virus (DENV) should be observed. Here, we evaluated a routinely used chikungunya commercial IgM (Immunoglobulin M) ELISA test (Anti-Chikungunya IgM ELISA, Euroimmun) to assess its performance in confirming chikungunya in a dengue endemic area. Samples (*n* = 340) representative of all four DENV serotypes, healthy individuals and controls were tested. The Anti-CHIKV IgM ELISA test had a sensitivity of 100% and a specificity of 25.3% due to the cross reactivities observed with dengue. In dengue acute cases, the chikungunya test showed an overall cross-reactivity of 31.6%, with a higher cross-reactivity with DENV-4. In dengue IgM positive cases, the assay showed a cross-reactivity of 46.7%. Serological diagnosis may be challenging and, despite the results observed here, more evaluations shall be performed. Because distinct arboviruses co-circulate in Brazil, reliable diagnostic tools are essential for disease surveillance and patient management.

## 1. Introduction

Arboviruses are a major threat to public health in general, especially in tropical and subtropical countries [1,2,3]. In Brazil, chikungunya was first detected in Bahia and Amapá, in 2014, and, in a short period of time, cases were reported all over the country. Over the last years, Brazil has also reported several dengue outbreaks, resulting in a hyperendemic scenario [2,4,5,6,7].

Although dengue virus (DENV) and chikungunya virus (CHIKV) belong to two different families, *Flaviviridae* and *Togaviridae*, respectively, they are mainly transmitted by the same mosquito vector, *Aedes aegypti* [8]. Moreover, those viral infections share many signs and symptoms, such as fever, headache, exanthema, arthralgia, myalgia, nausea, and vomiting, that are, sometimes, clinically indistinguishable [9]. Therefore, clinical-based chikungunya diagnosis is usually difficult, especially in areas endemic for dengue [10], such as the one in Brazil.

CHIKV infection is rarely fatal, but it can result in important joint and neurological sequelae [11]. Usually, after two weeks, those symptoms disappear, but a significant portion of patients can persist with a chronic illness for months or even years after the initial infection. Anti-CHIKV IgM indicates an active infection, is produced within 3–8 days after onset of symptoms and can persist up to 10 to 18 months [12,13]. Infection with any of the four DENV serotypes produces a wide spectrum of clinical manifestations, including mild to severe signs and symptoms that may evolve to a fatal outcome [14].

For laboratory diagnosis, RT-PCR is an excellent tool for early confirmation of CHIKV infections, and many protocols have been established for this purpose [15,16,17,18,19,20,21,22,23]. However, their performance depends on the viremic phase of the disease—up to 7 days after onset of symptoms, more expensive equipment and trained technical support is needed [12,24]. Although enzyme-linked immunosorbent assays (ELISA) are less expensive and easier to perform, existing serological tests cannot detect CHIKV infection in the early phase of the disease. Recently, a CHIKV antigen-based ELISA and a lateral flow test using high-affinity anti-CHIKV antibodies was developed for acute diagnosis of CHIKV infections and, despite being highly specific, the overall sensitivity was 51% [25]. In recent years, some commercial CHIKV diagnostic kits have become available for chikungunya serological diagnosis and have been evaluated [26,27,28]; however, evaluations on endemic regions such as the one in Brazil, where multiple arboviruses co-circulate, are scarce.

The clinical differentiation between DENV and CHIKV can be challenging, and is crucial for the patient’s management [9], especially considering the life-threatening outcome dengue may result in. Moreover, as both CHIKV and DENV are currently circulating in Brazil, co-infections may occur. In that scenario, a reliable laboratorial diagnosis is imperative. As chikungunya is an alphavirus, no serological cross-reactivity with the flaviviruses, such as dengue, should be observed, making diagnosis more straightforward [29]. Here, we aimed to evaluate a commercial IgM ELISA test, routinely used for chikungunya diagnosis in Brazil, and access its performance in confirming the CHIKV infection in a dengue-endemic area.

## 2. Materials and Methods

### 2.1. Ethics Statement

The cases analyzed in this study were from an ongoing project approved by the Oswaldo Cruz Foundation Ethic Committee (CAAE 57221416.0.1001.5248, approved on 14 February 2017). The patient’s personal information was anonymized before the data were accessed.

### 2.2. Clinical Samples

The serum samples analyzed in this study by RT-PCR and anti-chikungunya virus ELISA IgM (Euroimmun Medizinische Labordiagnostika, Lübeck, Germany) belong to a previously gathered serum collection of the Viral Immunology Laboratory at Oswaldo Cruz Institute, Oswaldo Cruz Foundation, FIOCRUZ, Brazil, from epidemics that occurred from 1998 to 2018.

A panel of 340 sera was divided into fifteen groups as follows: Groups A to D, acute sera (up to 7 days of symptoms) from patients infected with DENV-1 (*n* = 33), DENV-2 (*n* = 12), DENV-3 (*n* = 20) and DENV-4 (*n* = 30), respectively, confirmed by molecular detection and/or virus isolation, and Group E, sera from patients with dengue infection serologically confirmed by MAC-ELISA with negative virus isolation and RT-PCR (*n* = 60). All dengue samples were from confirmed cases that occurred during epidemics from 1998 to 2003, with the exception of DENV-4, collected in 2012 and 2013. Group F was composed of chikungunya IgM and/or IgG positive samples (*n* = 28) previously tested by the Euroimmun Anti-chikungunya virus IgM kit, as well as by the Anti-CHIKV IgG one (catalogue numbers EI 293a M and EI 293a G, respectively); Group G, by sera from healthy individuals (*n* = 22); Group H, sera from individuals negative for both dengue after serological (IgM and NS1) and molecular (RT-PCR) testing and chikungunya after serological (Euroimmun IgM and IgG ELISAs) and molecular (RT-PCR) testing (*n* = 52); Group I, sera from yellow fever positive individuals (*n* = 09); Group J, sera from individuals vaccinated for yellow fever (*n* = 07); Group K, sera from measles patients (*n* = 12); Group L, sera from rubella patients (*n* = 12); Group M, sera from zika patients (*n* = 16); Group N, sera from leptospirosis patients (*n* = 17), and Group O, sera from hepatitis C patients (*n*= 10).

The well-characterized dengue panel was based on the diagnosis of the cases by MAC-ELISA (Panbio dengue IgM Capture ELISA, E-DEN01M, Brisbane, Australia), IgG-ELISA, according to Miagostovich et al. [30], NS1-ELISA (Platelia^TM^ Dengue NS1 Ag-ELISA kit, Biorad Laboratories, Marnes-La-Coquette, France) and/or RT-PCR according to Lanciotti et al. [31] or Johnson et al. (2005) [32]. The zika positive cases were confirmed according to Lanciotti et al. [33]. Samples from cases positive for measles and rubella were kindly provided by the Respiratory Virus and Measles Laboratory, National Reference Laboratory, at Oswaldo Cruz Institute, FIOCRUZ, Rio de Janeiro, Brazil. Samples from cases positive for yellow fever and vaccinees were provided by the Flavivirus Laboratory, Regional Reference Laboratory, at Oswaldo Cruz Institute, FIOCRUZ, Rio de Janeiro, Brazil. Samples from patients positive for hepatitis C were provided by the Viral Hepatitis Laboratory, at Oswaldo Cruz Institute, FIOCRUZ, Rio de Janeiro, Brazil. Positive samples of leptospirosis were provided by the Laboratory of Bacterial Zoonosis, at Oswaldo Cruz Institute, FIOCRUZ, Rio de Janeiro, Brazil.

### 2.3. Anti-Chikungunya Virus IgM ELISA

The Anti-Chikungunya Virus ELISA IgM (Euroimmun, Medizinische Labordiagnostika, Lübeck, Germany, catalogue number EI 293a M) provides a semi quantitative in vitro assay for the detection of human anti-CHIKV IgM in serum or plasma for the diagnosis of chikungunya and it was performed according to the manufacturer’s instructions. Briefly, serum or plasma samples diluted 1:101 in sample diluent containing anti-human IgG were added to microtiter wells coated with a mixture of recombinant CHIKV antigens. After the wells sat for 1 h at 37 °C and were washed, peroxidase-labeled anti-human IgM was added. After 30 min at room temperature (RT), the wells were washed again and then reacted with a chromogen-substrate solution (tetramethylbenzidine (TMB) plus hydrogen peroxide) for 15 min at RT. The color reaction was stopped by the addition of 0.5 M sulfuric acid. Optical densities (ODs) were measured at 450 nm and 620 nm (reference wavelength) for each well using a spectrophotometer reader. Per the manufacturer’s instructions, which report a sensitivity of 98.1% and a specificity of 98.9%, the results were expressed as index values, calculated by dividing a specimen’s OD by the OD of a kit-supplied calibrator serum included in the same run. Index values of 0.8 were considered negative, values of 0.8 to 1.1 were considered equivocal, and values of 1.1 were considered as positive.

### 2.4. Statistical Analysis

Statistical analyses were performed using GraphPad Prism software, version 9.0 (GraphPad Software Inc., San Diego, CA, USA). The nonparametric Mann–Whitney U test was used to evaluate differences between groups. Values of *p* < 0.05 were considered significant for all statistical analysis.

## 3. Results

**Figure 1:**
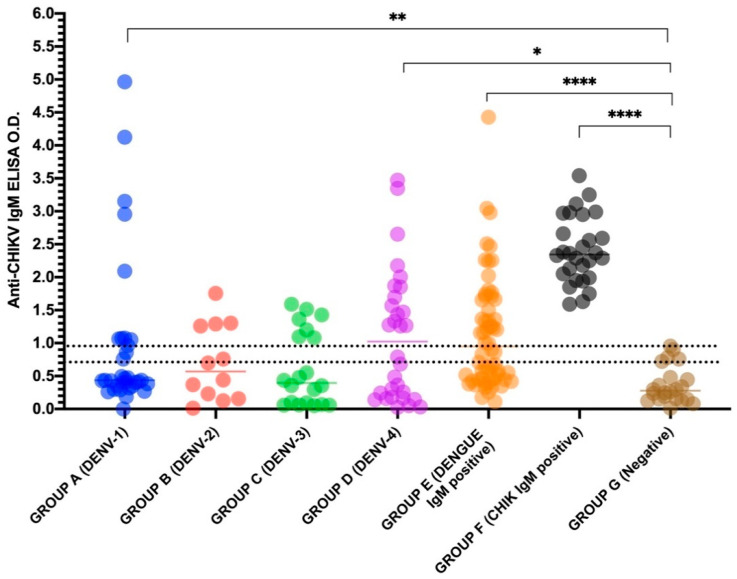
Absorbance values obtained using the Anti-chikungunya IgM ELISA test (Euroimmun) on a panel (*n* = 340) of dengue, chikungunya and controls. The nonparametric Mann–Whitney U test was used to evaluate differences between optical density (OD) among the dengue and chikungunya groups (Groups A–F) in comparison to the healthy individual group (Group G). **** *p* < 0.0005, ** *p* < 0.005, * *p* < 0.05, (-) represents the mean value for each group and (---) dashed lines, the cut-off interval for the test. The negative (<0.8) and positive (>1.1) cutoff values were determined at a wavelength of 450 nm and a reference value between 620 nm and 650 nm.

In dengue acute cases (Groups A–D), the anti-chikungunya virus IgM ELISA test showed an overall cross-reactivity of 31.6% (30/95), independently of the infecting serotype, with a higher cross-reactivity observed for DENV-4 cases (Group D; 50.0%), followed by DENV-2 (Group B; 33.3%). Furthermore, the absorbance values obtained for DENV-1 and DENV-4 were significant, when those were compared to the negative samples (Group G, *p* = 0.0027 and *p* = 0.0130, respectively), Figure 1.

In anti-DENV IgM positive samples (Group E), the anti-chikungunya virus IgM ELISA showed cross-reactivity in 46.7% (28/60) of the cases (Table 1), and the differences observed in the OD when those were compared to the negative samples were highly significant (*p* < 0.0001) (Figure 1).

Information on dengue immune response (Groups A–E, *n* = 155) was available in 27.7% (43/155) of the cases, and 53.5% (23/43) of those were characterized as primary dengue infections and 46.5% (20/43) as secondary ones (data not shown).

Aiming to avoid potential false positive results, except by the DENV-4 that was introduced in 2010 in Brazil, all other dengue cases analyzed here were collected during epidemics that occurred from 1998 to 2003, some of them more than ten years prior to CHIKV circulation in the country. Despite that, DENV-4 cases were also collected prior to the CHIKV circulation in Brazil.

In chikungunya positive cases (Group F), considered “true positive”, the commercial test was highly sensitive and confirmed 100% (28/28) of the cases tested, Table 1. The chikungunya positive cases analyzed here were previously tested by the same Euroimmun anti-chikungunya virus IgM kit, as well as by the anti-CHIKV IgG one (catalogue numbers EI 293a M and EI 293a G, respectively), during the 2018 epidemic in Rio de Janeiro, Brazil. Because those cases were retested with the same kit, they were included to check the test’s reproducibility and for comparison purposes. All cases (days 1 to 45 after the onset of symptoms) were anti-CHIKV IgM positive and eleven of them were also anti-CHIKV IgG positive. In this study, cases were retested by the anti-chikungunya virus IgM ELISA and again, resulted positive. Moreover, the differences on the absorbance values observed when those cases were compared to the negative group (Group G, healthy individuals) were statistically significant (*p* < 0.0001) (Figure 1).

The specificity for the anti-chikungunya virus IgM ELISA kit was 100% based on the analysis of sera from healthy individuals (Group G). However, on the sera of symptomatic suspected cases for arboviral infection (dengue), considered as negative after differential diagnosis for both dengue and chikungunya (Group H), the test was positive in 13.5% (7/52) of the cases. No cross-reactivity was observed with sera from patients infected with yellow fever (Group I) or those vaccinated against yellow fever (Group J), measles (Group K), rubella (Group L), zika (Group M), leptospirosis (Group N) and hepatitis C (Group O) (Table 1).

Overall, the anti-CHIKV IgM ELISA test had a sensitivity of 100%, with a specificity of 25.3% in all samples tested. In contrast, its negative predictive value was 100%, compared to the lower positive predictive value (30.1%), with a test efficiency of 43.5% (Table 2).

## 4. Discussion

The first autochthonous cases of chikungunya were confirmed in Brazil in 2014, when the virus was introduced, and simultaneously in Oiapoque (Amapá) and Feira de Santana (Bahia) [34]. The virus soon spread nationwide, causing epidemics in the following years. At the same time, the Brazilian territory was already dealing with a dengue burden, since its introduction in the 1980s [35,36]. Moreover, after the introduction of zika in 2015 [37,38], the country reported a triple epidemic of dengue, zika and chikungunya in 2016. From 2016 to 2018, more than 500,000 chikungunya suspected cases were reported in Brazil [39].

The simultaneous circulation of arborviruses leads to a challenging clinical-based diagnosis, due to the signs and symptoms shared. Therefore, a reliable laboratorial diagnosis is imperative for the disease surveillance and clinical management [10,40,41]. Serological techniques are widely available, easier to perform and relatively cheaper than molecular approaches [42]. Those tests allow the patients’ immune response to be investigated in a wider diagnostic window characterized by the detection of IgM antibodies, meaning an acute infection, and IgG antibodies meaning a previous viral exposure [12,40]. The IgM antibody capture ELISA (MAC-ELISA) that allows the detection of IgM antibodies in serum samples collected four days after the symptoms’ onset is the most commonly used test for the laboratory diagnosis of chikungunya [43], and it has been reported to be highly specific and highly accurate [28].

In a region where multiple arboviruses co-circulate, such as the one in Brazil, co-infections may occur [44,45,46,47,48,49]. Although both are associated with poor quality of life, evidence for more severe diseases in DENV/CHIKV co-infected individuals is poorly understood and controversial. According to Abhishek and Chakravarti, the co-infection further worsens the condition [50]. However, for Silva et al., there is no exacerbation of clinical signs [1]. Regardless, not only co-infections [44], but also the co-circulation of those arboviruses, is an additional challenge for the differential diagnosis.

Here, we report a cross-reactivity of dengue positive cases in a routinely used anti-CHIKV IgM ELISA test in Brazil. Overall, 37.4% (58/155) of the patients positive for dengue infection were also positive in the anti-chikungunya virus ELISA IgM (Euroimmun). Considering only the acute dengue cases (up to 7th day of symptoms, Groups A to D), the cross-reactivity was of 31.5% (30/95); however, for dengue IgM positive cases (Group E), this was even higher (46.7%). Moreover, CHIKV RNA was not detected by qRT-PCR when those cases were tested. Although arboviruses cocirculation and co-infections are common in Brazil and in the world nowadays [51,52,53,54,55], our dengue samples were collected in Rio de Janeiro between 1998 and 2010, when there was no report of CHIKV circulation in Brazil, with the exception of DENV-4 that was introduced in 2010 and cases were collected during the 2012–2013 epidemic. Reports of chikungunya fever in Brazil occurred in São Paulo in 2010 [56] and in Rio de Janeiro in 2012 [57], both imported cases from Indonesia.

Cross-reactivities in commercial immunoassays for arboviruses diagnosis have already been described. Felix et al. showed anti-DENV ELISA cross-reactivity with serum from ZIKV positive patients [58]. Zaidi et al. (2020) described how zika positive patients previously exposed to DENV showed higher levels of IgM anti-DENV than the DENV naïve patients when those were evaluated by widely used assays [59]. However, DENV and ZIKV belong to the same genus, *Flavivirus*, and CHIKV is an *Alphavirus*. Nevertheless, the Anti-CHIKV IgM ELISA test analyzed here showed no cross-reactivity with other control groups. Previous studies performed by CDC and another two public health agencies demonstrated a 98% average of sensitivity and specificity for anti-CHIKV IgM ELISA (Euroimmun). The Public Health Agency of Canada National Microbiology Laboratory, one of the study’s centers, used a panel of 147 samples to evaluate the Euroimmun IgM ELISA kit, and only 10 were from dengue, with no information on the infecting serotype. The Caribbean Public Health Agency reported a 100% accuracy of the considering the analysis of 37 samples, the sensitivity and specificity of which were 100% (26/26 and 10/10, respectively). However, the study reports some limitations, such as sample viability [28].

A recent study in Brazil evaluated both the Inbios and Euroimmun IgM-ELISA kits for the diagnosis of chikungunya in cases that occurred from 2014 to 2016, when DENV, CHIKV and ZIKV circulated simultaneously and, among dengue cases, specificities were 83.9% and 82.8% for the acute-phase samples, and 88.9% and 83.3% for the convalescent-phase samples, respectively. As expected, both tests sensitivities were higher on convalescent samples. DENV infection was identified only in 3.5% of the cases analyzed and no information on the infecting serotypes was provided. CHIKV/DENV co-infections were also reported in 1.3% of the cases. Moreover, the authors found that the Euroimmun IgM ELISA presented more equivocal results, with lower specificity than the Inbios test, resulting in a higher rate of false-positive cases in scenarios with low chikungunya prevalence [60].

Differently from Group G, composed by healthy individuals, Group H was composed of dengue-like symptomatic patients seeking assistance during epidemics that occurred from 1998 to 2010, but who were negative for dengue by serological (IgM, IgG and NS1 ELISAs) and molecular (RT-PCR) tests, and also negative for chikungunya by serological (IgM and IgG ELISAs) and molecular (RT-PCR) tests. In that group, seven cases showed cross-reactivity in the Euroimmun anti-Chikungunya virus IgM kit, but, despite that, the OD values observed (from 1.1 to 1.4) were near the kit’s cut-off (1.1) (data not shown). Which infection or condition led to those symptoms is unclear and not available, but whatever they were, they could have had some impact on the cross-reactivity observed in those cases.

Here, we analyzed a previously gathered serum collection from the Viral Immunology Laboratory, at FIOCRUZ, composed of samples collected during epidemics and routinely separated in at least five aliquots to avoid freeze–thawing during the investigations and testing. However, one limitation was the lack of prospective sampling in the analysis. Prospective cross-reactivity investigations shall also be performed on fresh sera, but considering current epidemiological scenarios, well-defined dengue, and dengue cases representative of four serotypes, may be troublesome to gather and compose a panel. Despite that, most evaluations studies, using well-characterized panels, do use retrospective sampling. A multicenter study performed by CDC to evaluate commercially available chikungunya IgM assays did use a panel of serum samples selected from the CDC Arboviral Diseases Branch collection of archived diagnostic specimens, and reported the importance of establishing and sustaining biobanks for assay evaluations [28].

In this study, the Euroimmun IgM-ELISA was highly sensitive but poorly specific, due to the cross reactivities observed with dengue cases. We did not include cases positive for other alphaviruses, as we aimed to evaluate the test performance for its use in a dengue endemic scenario. Dengue has been a major public health problem in Brazil for the past 30 years, and, after 2013, the country has been reporting more than a million cases almost yearly.

Arboviruses cocirculation in Brazil represents a challenge regarding diagnosis and clinical management, and frequently, dengue and chikungunya symptoms are similar, which can result in the diseases’ misdiagnosis. Therefore, an accurate and reliable diagnosis is needed for proper clinical care and surveillance. Nevertheless, despite the results observed here, more evaluations shall be performed where those viruses circulate.

## Figures and Tables

**Table 1:** Evaluation of the Anti-chikungunya IgM ELISA (Euroimmun) for chikungunya diagnosis based on the analysis of the distinct groups.

Groups	Sample Year	Anti-Chikungunya Virus IgM ELISA (Euroimmun) Positive/Tested (%)
A (DENV-1 cases, *n* = 33)	1998–2001	6/33 (18.2)
B (DENV-2 cases, *n* = 12)	1999–2010	4/12 (33.3)
C (DENV-3 cases, *n* = 20)	2001–2002	5/20 (25.0)
D (DENV-4 cases, *n* = 30)	2012–2013	15/30 (50.0)
E (Dengue IgM positive cases, *n* = 60)	1998–2003	28/60 (46.7)
TOTAL OF GROUP A–E (*n* = 155)		58/155 (37.4)
F (Chikungunya cases, *n* = 28)	2018	28/28 (100)
TOTAL OF GROUP F (*n* = 28)		28/28 (100)
G (Healthy individuals, *n*= 22)	2007–2018	0/22
H (Individuals negative for dengue and chikungunya; *n* = 52)	1998–2010	7/52 (13.5)
I (Yellow fever positive cases, *n* = 09)	1997–1998	0/09
J (Individuals vaccinated for yellow fever, *n* = 07)	1999–2018	0/07
K (Measles cases, *n* = 12)	2004–2005	0/12
L (Rubella cases, *n* = 12)	2005	0/12
M (Zika cases, *n* = 16)	2015	0/16
N (Leptospirosis cases, *n*= 17)	2009	0/17
O (Hepatitis C cases, *n* = 10)	2009	0/10
TOTAL OF GROUP I–O (*n* = 83)		0/83
TOTAL	1997–2018	93/340 (27.3)

**Table 2:** Overall performance of the anti-chikungunya IgM ELISA (Euroimmun) for chikungunya diagnosis in Brazil.

Performance * of the Anti-Chikungunya Virus IgM ELISA (Euroimmun)	%
Sensitivity	100
Specificity	25.3
Efficiency	43.5
Positive Predictive Value	30.1
Negative Predictive Value	100

* Sensitivity (a/a + b), specificity (d/c + d), efficiency (a + d/a + b + c + d), positive predictive value (a/a + c) and negative predictive value (d/b + d), where a = true positive (*n* = 28), b = false negative (*n* = 0), c = false positive (*n* = 65) and d = true negative (*n* = 22).

## Data Availability

All data generated during this study are included in this published article.
